# Systematic Review: Syndromes, Early Diagnosis, and Treatment in Autoimmune Encephalitis

**DOI:** 10.3389/fneur.2018.00706

**Published:** 2018-09-05

**Authors:** Christina Hermetter, Franz Fazekas, Sonja Hochmeister

**Affiliations:** Department of Neurology, Medical University of Graz, Graz, Austria

**Keywords:** autoimmune encephalitis, antibodies, surface antigens, clinical relevance, treatment

## Abstract

In recent years, new antibodies have been discovered which mediate autoimmune encephalitis. This immunological response can be triggered by an infection or a tumor. Classical onconeuronal antibodies are directed against intracellular neuronal agents but recently, a novel group of antibodies to neuronal cell-surface and synaptic antigens associated with different CNS-syndromes, has been discovered. Interestingly, the syndromes in this group can be successfully treated with immunotherapy and frequently do not have underlying tumors. The aim of this review is to describe the current state of knowledge about autoimmune encephalitis, in order to provide clinicians with a concise, up-to-date overview. Thus, a comprehensive literature search was performed in medical databases. The literature was carefully studied and new findings focusing on the symptoms, diagnosis and treatment were summarized and interpreted. Even though it might be challenging in some cases, the awareness of certain symptom constellations and demographic information, in combination with laboratory- and MRI-results, allows clinicians to make the diagnosis of probable autoimmune encephalitis at an early stage. Treatment can therefore be initiated faster, which significantly improves the outcome. Further investigations could define the underlying pathogenic mechanisms. Randomized controlled trials, paired with increasing clinical experience, will be necessary to improve the identification of affected patients, treatment strategies, and outcomes in the years to come.

## Introduction

Autoimmune encephalitis (AIE) may be associated with the presence of specific autoantibodies. In cases in which an autoantibody is detected in the CSF or serum, AIE can be divided into two groups, depending on the localization of the target antigen. In addition to the “well”-defined classical paraneoplastic syndromes with antibodies which target intracellular proteins (e.g., anti-Hu, anti-Yo, anti-Ri), a new group of antibodies associated with AIE and their correlating symptoms, have been defined. They interact directly with cell-surface neuronal receptors or synaptic proteins (1, 2). In the case of classical, paraneoplastic syndromes, the disease is triggered by an anti-tumor immune reaction and is considered to result primarily from a CD8+T-cell response with the antibodies being likely to arise secondarily to the cellular T-cell-driven damage directed at the intracellular molecules. The new group of antibodies against surface antigens, seems to be directly pathogenic and may change their target's structure or function, with resulting consequences on its behavior or tissue destruction, by receptor internalization or blockage, redistribution from the synaptic to the extra synaptic site, or interference with the ligand-receptor interaction. But the underlying causes for the pathogenic pathways leading to the antibodies accessing the CNS and the associated immune response are, as yet, poorly understood. The role of T-cells has not yet been fully established in detail (3, 4). Different mechanisms have been proposed, one including various infectious triggers which “prime” the immune system by activating T or/and B cells against similar epitopes by way of molecular mimicry, as in the case of Herpes simplex virus-encephalitis associated with NMDA-R-encephalitis (5, 6). Unlike in classical paraneoplastic syndromes, in cell-surface or synaptic antibody-syndromes the presence of a tumor is variable, they respond to multimodal immunotherapy and seem to have a better overall prognosis (7, 8). Awareness and knowledge is emerging rapidly through clinicians, due to a large number of case reports, as well as the performance of retrospective data analysis. However, in many cases, the diagnosis and the treatment remain challenging.

In this review we therefore focus on the clinical perspective of the symptoms, essential aspects of an early diagnosis and differential diagnosis, as well as the treatment options in adults.

## Characteristic clinical syndromes

Initially, the clinical features of different types of AIE may overlap. The symptoms include epileptic seizures, movement disorders, psychiatric, and cognitive alterations (9).

### AIE associated with antibodies against neuronal cell surface antigens

#### NMDA-R encephalitis prototype

To date, the best recognized subtype is N-methyl D-aspartate-receptor (NMDA-R) encephalitis. About 80% of the patients are young and female (median 20 years). Typically, symptoms emerge in stages. Patients usually develop virus-like prodromal symptoms, with headaches, lethargy and fever, followed by progressive behavioral changes, memory deficits, confusion, and psychosis within 2 weeks.

This progresses to language problems, epileptic seizures, a range of movement disorders and eventually, global encephalopathy, and dysregulation of autonomic functions may occur, with severe complications such as hyperthermia, cardiac arrhythmias, blood pressure instability, or coma (due to decreased NMDA-R influence in the brainstem), requiring intensive care unit management (2, 10). tumors can be found in one third of the patients. Women of reproductive age are mainly affected due to an ovarian teratoma while, in the elderly, it is more often a carcinoma. Other tumors that have been described are rare and include neuroblastoma, Hodgkin lymphoma, tumors of the breast, thymus and lung (10). Most of the patients require long-term hospitalization and subsequent rehabilitation. Depending on the early diagnosis, beginning of full treatment and time to tumor removal, full recovery usually takes up to 18 months. One group reported 75% with a modified ranking scale from 1 to 2, in a cohort of 360 patients, in which symptoms like memory or language deficits seemed to be the last to recover (10).

The limbic system is a predilection in autoimmune encephalitis and is the most consistently affected structure. Target proteins associated with classical limbic encephalitis are AMPA, GABAb, LGI1, and GAD. Additional clinical findings may allow further differentiation between these different types (11, 12).

**Anti-AMPA-R** (α-amino-3-hydroxy-5-methyl-4-isoxazolepropionic acid receptor) encephalitis typically progresses rapidly but in some cases, only psychiatric symptoms are present. Patients have a high risk of underlying tumors (lung, breast or thymus) (10). Immunotherapy is often successful initially but relapses occur frequently (1).

Patients with **anti-GABAb** encephalitis have a high association with neoplasms, including small-cell lung cancer or neuroendocrine tumors (11). Additional characteristic features are early and frequent prominent seizures or status epilepticus. Some patients might also exhibit ataxia and opsoclonus-myoclonus-syndrome. The syndrome usually responds well to immunotherapy (1, 10).

In addition to the symptoms of classical, limbic encephalitis, common features of leucine-rich glioma-inactivated protein 1(**LGI-1**) encephalitis are hypernatremia and rapid-eye-movement sleep disturbance. Prior to the encephalitis syndrome, patients frequently have highly repetitive, unilateral faciobrachial dystonic seizures (FBDS). The seizures are often refractory to anticonvulsive treatment but improve with immunotherapy. In comparison to NMDA-R-encephalitis, patients with LGI1-encephalitis usually seem to respond faster at the beginning of immunotherapy, although the long-term outcome tends to be less favorable. tumors known to be associated with LGI1-encephalitis are bronchial carcinoma and thymoma (1, 7, 10).

Like GABAb, the **GABAa**-antibody type has a high risk of severe seizures or often intractable status epilepticus, requiring pharmacologically induced coma (8, 10). The MRI often shows hyperintense lesions outside the limbic system, in contrast to all other forms (11).

Antibodies directed against **Glycine receptor** (Gly-R) and **DPPX** (dipeptidyl-peptidase-like protein-6) have been described in patients with brainstem and spinal cord hyperexcitability disorders, such as PERM-syndrome (progressive encephalomyelitis with rigidity and myoclonus). Gly-R antibodies were also found in a few cases of stiff-person-syndrome. Prodromal diarrhea with substantial weight loss is commonly reported in the DPPX- group (1). DPPX is also expressed in the myenteric plexus (13).

It is important to differentiate anti-contactin-associated protein-2 (**CASPR2**) encephalitis from motor neuron diseases. In rare cases, it is also associated with limbic encephalitis. It is more commonly associated with Morvan syndrome, a rare disease combining peripheral nerve hyperexcitability, neuromyotonia, autonomic disturbance and sometimes encephalopathy. Neuromyotonia is often associated with painful peripheral neuropathy but bulbar weakness can also occur. The same associated tumor entities have been described as for the LGI1-type (7, 10, 13).

Antibodies against metabotropic glutamate receptor 5 (**mGLUR5**) have been found in patients with Hodgkin lymphoma and Ophelia syndrome (limbic encephalitis with predominate memory deficits) and antibodies against mGLUR1, in cerebellar ataxia. Immunotherapy is often successful and full recoveries are achieved (1, 13).

The **Adenylate-kinase 5** antibody syndrome usually presents with isolated, severe short-term memory loss. There is no association with cancer but the response to immunotherapy is poor (14).

### AIE associated with antibodies against intracellular antigens

This group includes the classical onconeuronal antibodies (e.g., anti-Hu, Ri, Yo, Ma2/Ta and Amphiphysin) with their well-characterized syndromes, which will not be discussed any further here (for a comprehensive overview we refer to excellent review articles e.g., *Paraneoplastic neurological syndromes and autoimmune encephalitis* Stich and Rauer, 2014) and the glutamic acid decarboxylase 65 (GAD) antibodies.

High antibody titres against **GAD65**, a non-paraneoplastic intracellular antigen, are associated with different neurological symptoms including limbic encephalitis, seizures and cerebellar ataxia. They are also common in stiff-person-syndrome. There is usually no underlying tumor. Low titres of GAD antibodies can also occur in healthy and in up to 80% of type 1 diabetes mellitus patients (14).

Some of the neuronal antibodies are associated with concurrent thyroid antibodies. Thyroid antibodies are not specific and can also be present in 13% of healthy individuals. SREAT (Steroid-responsive encephalopathy with autoimmune thyroiditis) might be a differential diagnosis at disease onset, since the symptoms can be similar, but it can ultimately be excluded by the detection of neuronal surface antibodies (14).

Acute disseminated encephalomyelitis (ADEM) could be associated with neuronal antibodies and is an important differential diagnosis, as clinical and/or MRI findings can overlap. In an epidemiological study of the prevalence of autoimmune encephalitides, it was shown that MOG antibodies were among the neuronal autoantibodies with high specificity and were one of the most commonly detected (15). MRI can help to further differentiate ADEM from other autoimmune encephalitis particularly in the follow up, as ADEM should show no new clinical or MRI findings 3 months after the onset of symptoms (see *Diagnostic criteria for ADEM* Graus et al, 2017). In addition, cases of autoimmune encephalitis combined with demyelinating disorders have been reported many times. Therefore, patients with atypical features like optic neuritis or demyelination in MRI or otherwise prominent neuropsychiatric symptoms, should be tested for concurrent disorders (AQP4- and MOG antibodies plus NMDA-R antibodies) (14, 16, 17).

Other antibodies associated with encephalitis are listed in Table 1, for the purpose of completeness.

**Table 1:** Antibodies related to autoimmune encephalitis [onconeuronal antibodies are excluded; summarized from (7, 14, 18)].

**Antibody**	**Syndromes**	**MRI: T2/Flair Sequences**	**Tumor**	**F/M**	**Age (Median)**
NMDA-R	Prodromal stage, global encephalopathy	Normal or transient non-region specific changes (~33%)	10–50%, (age dependent) ovarian teratoma	4:1	1–85 (21)
LGI1	Faciobrachial dystonic seizures, limbic encephalitis, hyponatremia, sleep disorders, myoclonia	Hyperintense signal in medial temporal lobes and basal ganglia (>80%)	<10–20% Bronchial carcinoma, thymoma	1:2	30–80 (60)
AMPA-R	Limbic encephalitis (predominant psychosis), seizures	Hyperintense signal in medial temporal lobes (90%)	70% Bronchial- or Mamma carcinoma, Thymoma	9:1	38–87 (60)
GABAb-R	Limbic encephalitis, seizures	Hyperintense signal in medial temporal lobes (>60%)	60% Bronchial carcinoma, neuroendocrine tumors	1:1	24–75 (62)
CASPR2	Morvan syndrome, neuromyotonia, polyneuropathy, bulbar weakness, limbic encephalitis	Normal or Hyperintense signal in medial temporal lobes (~40%)	<20–40% bronchial carcinoma, thymoma	1:4	46–77 (60)
Glycine-R	PERM, Myelopathy, Stiff person syndrome	Normal or nonspecific changes (~10%)	~10% Lymphoma, thymoma	6:5	5–69 (43)
mGLUR5	Ophelia syndrome	Normal or hyperintense signal in various brain regions (~50%)	Hodgkin lymphoma	1:2	35
GAD*	Stiff person syndrome, limbic encephalitis, seizures, cerebellar ataxia	n/k	25% Thymoma, small cell lung carcinoma	n/k	n/k
GABAa-R	Encephalitis with refractory seizures	Hyperintense signal in multiple cortical and subcortical regions	25% Thymoma	n/k	n/k
DPPX	Encephalitis, diarrhea, hyperplexia	Normal or nonspecific changes	<10% Lymphoma	n/k	n/k
Dopamine-2-R	Basal ganglia encephalitis with abnormal movements, gait disturbance	Hyperintense signal in basal ganglia	n/k	1:1	2–15 (6)
Neurexin-3 α	Encephalitis	Normal	n/k	n/k	n/k
IgLON5	NREM and REM sleep disorder, brain stem dysfunction	Normal	n/k	n/k	n/k
mGLUR1	Cerebellar ataxia	Normal or cerebellar atrophy	A few cases described, Hodgkin disease	n/k	n/k
nACH-R	Encephalitis, postural tachycardia syndrome, Chronic intestinal pseudo-obstruction	Not applicable	30% thymoma, mamma/bladder/rectum/bronchial carcinoma, lymphoma	2:1	20–76 (58)
MOG	Acute disseminated encephalomyelitis	Diffuse, poorly demarcated, large (>1–2 cm) lesions predominantly in the white matter	0%	n/k	n/k
Adenylate-kinase 5	Isolated severe short-term memory loss, no seizures	Not applicable	No association	n/k	n/k

* *Intracellular target, all other antibodies listed have cell-surface/synaptic targets*.

*Abb.:n/K not known*.

**Table:** 

**Diagnostic criteria for possible autoimmune encephalitis**
subacute onset (usually within a few weeks but less than 3 months) with change in personality or level of consciousness and symptoms suggesting involvement of the limbic system including working memory deficits, psychiatric symptoms and seizures. At least one of the following: - A new focal clinical CNS event - EEG with epileptic or slow-wave activity - CSF pleocytosis - MRI findings suggestive of encephalitis^*^ Reasonable exclusion of alternative causes^**^.

* Hyperintense signal on T2-weighted/FLAIR highly restricted to one or both medial temporal lobes or in multifocal areas involving gray or white matter compatible with demyelination *or* inflammation (see below),

** CNS infections, septic encephalopathy, metabolic encephalopathy, drug toxicity, cerebrovascular disease, neoplastic disorders, Creutzfeldt-Jakob disease, epileptic disorders, rheumatologic disorders, mitochondrial diseases [summarized from (14, 17, 21)].

**Table:** 

**Diagnostic criteria for definite autoimmune encephalitis**
Subacute onset (usually within a few weeks but less than 3 months) with change in personality or level of consciousness and symptoms suggesting involvement of the limbic system including working memory deficits, psychiatric symptoms and seizures. At least one of the following: - EEG with epileptic or slow-wave activity - CSF pleocytosis. Typical MRI findings: Bilateral hyperintensities on T2-weighted/FLAIR sequence highly restricted to the medial temporal lobes. Reasonable exclusion of alternative causes.

*If all of the above criteria match, the definitive diagnosis can be made (14)*.

**Table:** 

**Diagnostic criteria for autoantibody-negative possible autoimmune encephalitis**
Rapid progression (less than 3 months) of working memory deficits, psychiatric symptoms, altered mental status. At least two of the following: - MRI findings suggestive of encephalitis - Brain biopsy showing inflammatory infiltrates and excludes other disorders - CSF pleocytosis, CSF-specific oligoclonal bands and/or elevated CSF IgG Index. Exclusion of well-defined syndromes of autoimmune encephalitis (e.g., ADEM, Bickerstaff's encephalitis). Reasonable exclusion of alternative causes.

*If all of the above criteria match, the definitive diagnosis can be made (14)*.

## Diagnosis

The clinical diagnosis of AIE can be challenging. Initially, the symptoms of different types of AIE can overlap. Occasionally, combinations with headaches, mild hyperthermia, and frequent CSF pleocytosis, can also mislead to empiric antiviral or antibiotic treatment until the results for infectious encephalitis are completed, or the diagnosis is delayed by the resemblance to psychiatric illnesses (1, 8). Psychiatric disorders are generally the most common symptoms at AIE onset (19). Their symptoms are multiple and nonspecific; psychotic symptoms, hallucinations, paranoid thoughts, catatonia, behavioral and mood disorders can be present and can change during the course of the disease. Physicians in general need to be aware of this and initiate accurate diagnosis and treatment early on. Also, the benefits of psychotropic drug treatment are very limited in cases of AIE. Some medications, especially first-generation antipsychotics, might be even harmful (20).

A careful history taking may be helpful, as prodromal symptoms often occur (1, 8).

The following symptoms suggests a “probable” autoimmune encephalitis before antibody detection: A combination of characteristic clinical features in most cases but with different severity or dominance, together with additional information and specific findings such as age and gender, specific movement disorders (e.g., facial-brachial dystonic seizures), accompanied comorbidities (hyponatremia, diarrhea, and work-up or history for tumor), neuroimaging findings or EEG patterns, good empiric treatment response and no reasonable alternative diagnosis (1, 11). Antibody detection is unlikely to be an early diagnostic criterion because results take several days at least and are not available at disease onset. It is a confirmatory diagnostic test, however, the test can also be negative in up to 50% of autoimmune encephalitis series (14, 17).

Possible complications include coma, hyperkinesia (injuries, ventilation problems), autonomic dysfunction and prolonged need for artificial respiration and intensive care treatment (2).

The most important differential diagnosis to rule out is infectious encephalitis. If the clinical suspicion is high, an initially negative PCR should be retested (e.g., HSV PCR can be negative when tested within 24 h of onset) (14). Other differential diagnoses include metabolic or endocrine encephalopathies, psychiatric disorders, malignant neuroleptic syndrome, and rheumatic diseases (Sjögren-Syndrome or Lupus) (2).

The following diagnostic criteria have been reviewed and updated by a panel of experts in autoimmune encephalitis and should guide clinicians in making an early diagnosis, which is not dependent on the autoantibody status. In order to further classify the subtype with comorbidities, the malignancy association, and prognosis of autoantibodies remain crucial (14).

### Obligatory diagnostic tools

At onset of the symptoms, CSF and serum analysis show a mild to moderate lymphocytic pleocytosis (< 100 cells/μl) in 60–80% of patients. One third of patients show mild to moderate increased protein concentration, and 50% of patients show oligoclonal bands (1, 14). However, unremarkable CSF findings do not exclude the diagnosis (22).

The sensitivity of antibody testing has only been investigated in a few types of AIE, primarily in NMDA-R-encephalitis. Different studies have shown that patients with NMDA-R encephalitis had no detectable antibodies in serum in 14% of cases, but they all had them in CSF. The situation seems to be similar for other autoantibodies. LGI1-encephalitis is exceptional and often shows normal CSF findings (1, 6, 14).

Therefore, both serum AND CSF should always be tested for antibodies.

Antibody titres may correlate with clinical severity, but determining the clinical relevance of an antibody based on the titre is not recommended. The clinical picture and additional comorbidities are more reliable for evaluating a treatment response, course and prognosis (23).

The IgG-antibody subtype is classified as being pathogenic in most of the established syndromes. IgA and IgM-antibodies have unclear significance and have also been described in many other psychiatric disorders and in healthy controls (8).

In summary, antibody testing can never replace clinical judgement; the finding of antibodies *only* in serum or non-IgG-isotypes together with an atypical clinical picture for the identified antibody, should be interpreted cautiously (21).

Clinicians need to be aware of the pitfalls in antibody testing, like those mentioned above in respect of GAD antibodies and thyroid antibodies. Another example are the voltage-gated potassium channel (“VGKC”) antibodies. VGKC complex antibodies were the first surface receptor antibodies associated with AIE to be detected. Recent studies have differentiated LGI1 and Caspr2 antibodies related to VGKC complex antibodies. They are associated with a limited subset of syndromes, whereas other VGKC antibodies have an unknown specificity and might occur in any type of cell damage. They are also described in patients with non-autoimmune, pre-existing conditions. One group investigated the clinical relevance of VGKC positivity in patients without LG1 and Caspr2 antibodies in comparison with VGKC negatives, according to clinical criteria, in order to determine evidence for autoimmune inflammation in both groups. When antibodies to LG1/Caspr2 were lacking, there were no differences between the groups, implying that VGKC positivity is not a clear marker for autoimmune-mediated pathogenesis and does not contribute to diagnosis in clinical practice (4, 24).

**Figure 1:**
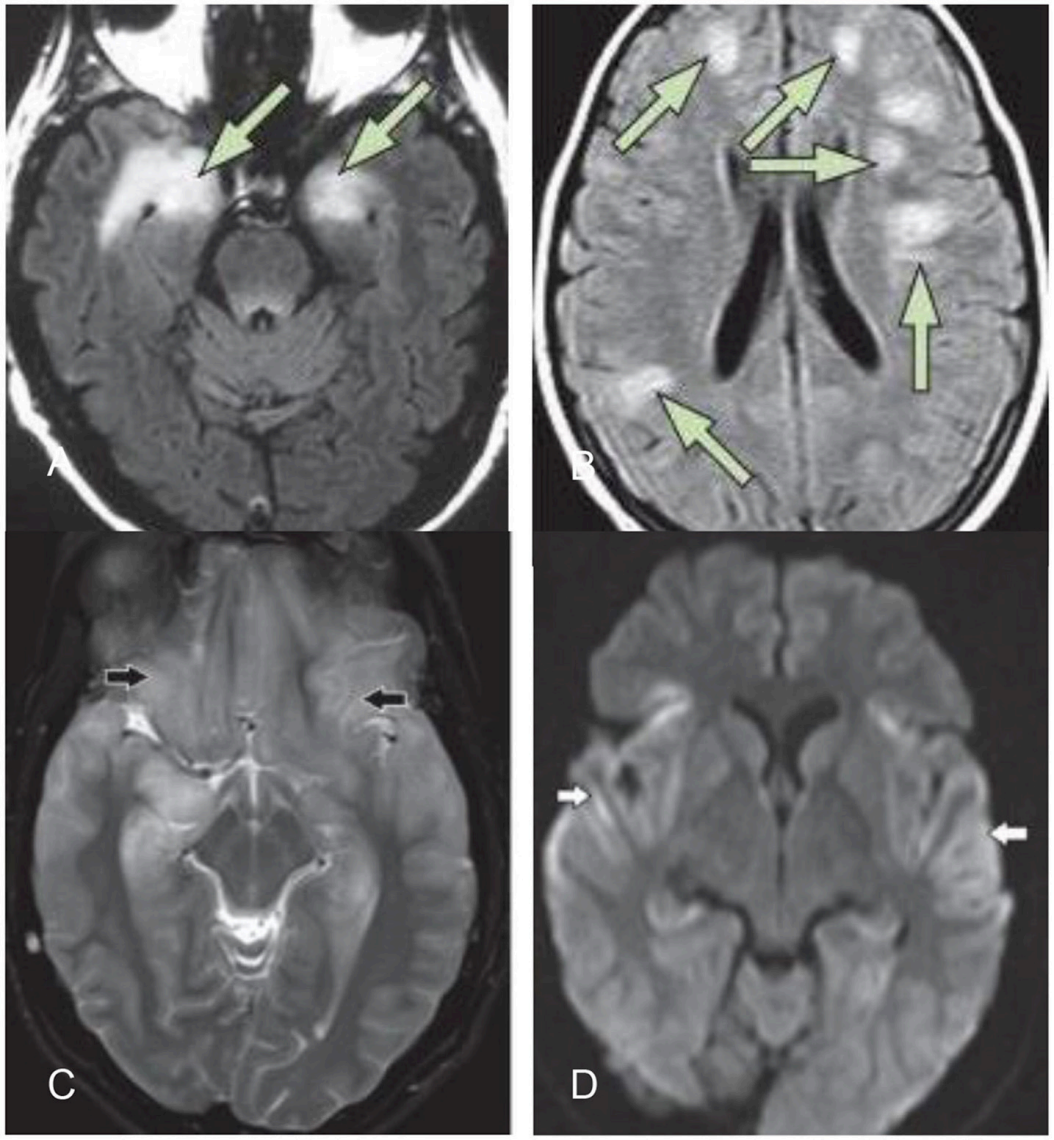
**(A)** Typical MRI of limbic encephalitis with bilateral hyperintensities in the medial temporal lobe on T2-weighted fluid–attenuated inversion recovery imaging **(B)** typical MRI of ADEM (14). **(C,D)** Herpes simplex virus encephalitis: bilateral symmetric cortical swelling and hyperintensity on T2 weighted image involving the anteromedial temporal lobes, insular cortex, orbital gyri (black arrows) with restricted diffusion in the involved areas (white arrows) (25).

MRI is frequently normal or shows only slight alterations. Common findings are not specific but typically show high uni- or bilateral T2/Flair-signals, especially in the medial temporal lobe with extrahippocampal cortical or subcortical lesions, without restricted diffusion or hemorrhage (11). A definitive distinction between infectious (e.g., HSV, tuberculosis) and autoimmune encephalitis is usually not possible from MRI alone (7, 8, 17). HSV encephalitis is less restricted to the limbic system and often shows restricted diffusion abnormalities and contrast uptake (14). In the case of LG1 encephalitis, hyperintensities in the basal ganglia are common (22). Figure 1 shows common MRI findings in different types of encephalitis.

The EEG is often abnormal. Apart from extreme delta brush (a generalized rhythmic delta activity with superimposed fast activity), which if present, is characteristic for NMDA-R-encephalitis, there are no pathognomonic patterns (8). Unspecific, frequent findings include general slowing, epileptic potentials or status and periodic lateralized epileptiform discharges (PLEDs) (17).

Tumor screening is essential. The range and frequency of associated malignancies differs according to the autoantibody detected. Depending on the autoantibody or clinical syndrome, specific tests (ultrasound, CT or MRI) should be performed based on their sensitivity (8). All patients may need chest, abdomen and pelvic-CT scans. Females should also undergo gynecological exams, breast, and ovarian ultrasound, and if negative, pelvic MRI for small teratomas. Males should undergo urological evaluation and ultrasound. Positive antibody-detection is highly associated with malignancies in older patients (>60 years). If CT/MRI and ultrasound do not yield any findings, a whole-body 18F-Fluorodeoxyglucose (FDG)-PET should be considered (17, 22, 26).

PET neuroinflammation imaging might play an important role in the future, as new radiotracers are currently being developed in clinical studies; their potential in assessing neuroinflammation still requires evaluation but might provide deeper insights into the complex immunopathology (27, 28).

The early detection of tumors is important, not only for the prognosis but also as treatments with immunotherapy could complicate tumor detection (e.g., lymphoma) (8).

### Infections as possible triggers

Herpes-simplex-encephalitis (HSE) is usually monophasic. Nevertheless, around 25% of cases that have been successfully treated with antiviral therapy show relapse after several weeks. In some cases, this might be due to viral reactivation but in others, especially those which presented with new symptoms, the new CSF samples showed NMDA-R antibodies without viral reactivation and the symptoms resolved after immunotherapy. Therefore, HSE patients that worsen after resolved infectious encephalitis, should be tested for infectious and autoimmune encephalitis (12).

Untreated Campylobacter jejuni infections can induce ganglioside-autoantibody mediated diseases, including Guillain-Barré-syndrome, Miller-Fisher-syndrome and in the CNS, Bickerstaff encephalitis. Characteristic findings are subacute onset, progressive impairment of consciousness, ataxia, and ophthalmoplegia. MRI shows brainstem abnormalities in 23% of cases, VGKC-antibodies may be present but are uncharacteristic findings and also frequently detectable in non-autoimmune diseases. Anti-GQ1b antibodies are confirmatory and make a clear distinction possible (5, 14).

## Treatment and prognosis

There are currently no randomized, controlled trials based on standard immunotherapy protocols, however, many retrospective and some prospective studies have clearly suggested the efficacy of immunomodulatory therapy. Seventy percent of patients respond to gradual immunotherapy escalation. Co-existing tumors, age, and delay in treatment are additional factors which determine the outcome. In general, young patients have a better outcome (5, 29).

First line therapy consists of corticosteroids plus IVIG and/or plasma exchange (PLEX)/immunoadsorption. Previous studies have shown that the use of high-dose corticosteroids is initially associated with better clinical outcome. In contrast to steroids, IVIG, and plasmapheresis/immunoadsorption are unlikely to worsen infectious encephalitis. In cases where there is a reasonable suspicion of autoimmune encephalitis, a multimodal immunological treatment may be started prior to CSF-antibody results, especially when the MRI findings reinforce the diagnosis (2, 8).

Although in many studies corticosteroids appear to be effective in AIE, the largely antibody-mediated disease pathogenesis needs to be considered. The effect of corticosteroids on B cells and Igs is limited and additional treatment may be required (30).

So far, there is no strong evidence of a difference in efficacy between IVIG and plasmapheresis. It must be considered in the therapy plan that IVIG can be removed by PLEX. Therefore, PLEX immediately after IVIG therapy is not recommended (22).

Selective immunoadsorption represents another extra-corporal antibody depletion method which has been proven in a few clinical studies to be effective as part of the multimodal immunotherapy of AIE, leading to clinically relevant improvement. Compared to PLEX, immunoadsorption allows a more targeted removal of proteins and avoids the disadvantages of plasma substitution (e.g., risk of infection or allergic reactions) and the impact on coagulation. All coagulation factors were significantly reduced by 50–70% after PLEX, whereas after immunoadsorption, single factors were not or only moderately, reduced. Documented side effects of immunoadsorption were nonspecific and related to intravenous lines (22, 31).

IVIG is more convenient for the patient and is cost-effective, compared to invasive options for antibody depletion. It is also more readily available for immediate therapy.

If there is little or no clinical improvement, second-line therapy should be implemented with Rituximab or Cyclophosphamide, with the former having a favorable side effect profile (8, 21). Different immunosuppressing drugs can be considered for long-term treatment (7). As, so far, there is no evidence to suggest the superiority of any specific regimen, Table 2 provides an overview of the immunomodulatory treatments and possible adverse effects, to support clinicians in the decision-making process for the individual patient.

**Table 2:**
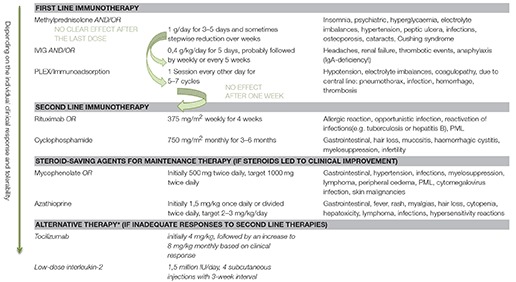
Immunomodulatory agents, dosing regime, and adverse effects.

*No clear recommendation! There are observational studies that resulted in clinical improvement with this alternative therapy, but these results remain to be confirmed, more evidence is needed before making final conclusions (30).

The optimal duration of these treatments is unknown. The clinical picture and issue of relapse rates (e.g., known high relapse rates in LGI1-R-encephalitis) should be considered and might lead to a longer or continuous treatment with Rituximab or Cyclophosphamide. Relapses should be treated with the same treatment scheme as the first clinical presentation (2).

In tumor-associated autoimmune encephalitis, surgical treatment should be initiated as soon as possible. It can relieve the symptoms effectively and favors the long-term outcome (2). A worsening of symptoms could, in contrast, suggest an incomplete resection, recurrence or secondary metastasis (5, 21).

Seizures may be very difficult to control and pharmacologically induced coma is frequently needed until the autoimmune disease regresses (6). So far, there is no evidence that one anticonvulsive medication is more efficacious than others. Due to the neuropsychiatric side effects of Levetiracetam, it might be difficult to determine whether, for example, acute agitation is due to the disease or pharmacologically reinforced. Lamotrigine, Benzodiazepines and Lacosamide can be used, as they do not seem to have a strong impact on cognitive function (22).

Follow up measurement of antibody titres during therapy, especially in serum, are not useful for treatment decisions, as they can test low in the initial analysis, even if the patient is in a coma. They can persist for years, even when the patient has fully recovered. However, in cases of relapses, it might be helpful to determine the course of the antibody titre (1).

Overall, encephalitis cases associated with surface-antibodies have a better prognosis than those associated with intracellular antibodies. However, in all cases, early stage treatment is crucial (1, 2).

Clinicians need to be aware that neurological symptoms may appear a long time before a tumor is detectable (e.g., micro teratomas), therefore, if the initial screening was negative, repeated follow-ups must be performed. Surveillance imaging at intervals of 4 to 6 months for at least 4 years are suggested (7, 17).

## Conclusion

The recognition of certain symptom constellations is crucial. When patients present with a clinical picture of encephalitis or sudden altered mental state, it is extremely important to consider an underlying autoimmune pathogenesis early on. If the listed criteria support the diagnosis of possible autoimmune encephalitis, treatment can be implemented early and prior to the onset of severe complications. Once the antibody results are available, the treatment can be re-evaluated and adapted.

The underlying mechanisms for activation and autoimmune response in the CNS are still unclear. Further investigations are needed to gain sufficient insights into how immune mechanisms affect nervous system functions. In addition, randomized, controlled trials could help to establish more specific therapies for the different subtypes of AIE.

## Author contributions

CH and SH wrote the manuscript, FF contributed by helpful discussion. All authors have read and approved the manuscript.

### Conflict of interest statement

The authors declare that the research was conducted in the absence of any commercial or financial relationships that could be construed as a potential conflict of interest.
